# 3D Human Periodontal Stem Cells and Endothelial Cells Promote Bone Development in Bovine Pericardium-Based Tissue Biomaterial

**DOI:** 10.3390/ma12132157

**Published:** 2019-07-05

**Authors:** Jacopo Pizzicannella, Sante D. Pierdomenico, Adriano Piattelli, Giuseppe Varvara, Luigia Fonticoli, Oriana Trubiani, Francesca Diomede

**Affiliations:** 1 Department of Medical, Oral and Biotechnological Sciences, University “G. d’Annunzio” Chieti-Pescara, 66100 Chieti, Italy; 2 ASL02 Lanciano-Vasto-Chieti; “Ss. Annunziata” Hospital, 66100 Chieti, Italy; 3 Chair of Biomaterial Engineering, Catholic University of San Antonio of Murcia (UCAM), 30001 Murcia, Spain; 4 Villa Serena Foundation for Research, 65013 Città Sant’Angelo (Pescara), Italy

**Keywords:** human Periodontal Ligament Stem Cells, Endothelial Cells differentiation, Vascular Endothelial Growth Factor-A (VEGF-A), Runt-related transcription factor 2 (RUNX2), bovine pericardium membrane

## Abstract

Bone defects repair represents a public and urgent problem in clinical practice, in fact, every year, more than two million patients required new treatments for bone injuries. Today a complete vascularization is strategic in bone formation, representing a new frontier for clinical application. Aim of this research has been developed a three-dimensional (3D) coculture platform using a bovine pericardium collagen membrane (BioR) loaded with human periodontal ligament stem cells (hPDLSCs) and endothelial differentiated cells from hPDLSCs (E-hPDLSCs) able to undergo toward osteoangiogenesis differentiation process. First, we have characterized at confocal laser scanning microscopy (CLSM) level the E-hPDLSCs phenotype profile, through CD31 and CD34 markers expression and the ability to tube vessel formation. Real Time-Polimerase Chain Reaction (RT-PCR) and western blotting analyses revealed the upregulation of Runt-related transcription factor 2 (RUNX2), Collagen 1A1 (COL1A1), Vascular Endothelial Growth Factor-A (VEGF-A) genes and proteins in the living construct composed by hPDLSCs + E-hPDSCs/BioR. Human PDLSCs + E-hPDLSCs/BioR construct showed also an enhacement of de novo synthesis of osteocalcin. Given that, the extracellular-signal-regulated kinase (ERK)/mitogen activated protein kinase (MAPK) transduction signaling was involved in the osteogenesis and angiogenesis process, the ERK1/2 protein level at biochemical level, in our experimental model, has been investigated. Our results evidenced an upregulation of ERK1/2 proteins level born in the living construct. In conclusion, we believe that the use of the hPDLSCs and E-hPDLSCs coculture togheter with BioR as substrate, could represent an efficient model able to activate through ERK1/2 signaling pathway the osteoangiogenesis process, and then representing a new potential engineered platform for surgeons during the repair and the healing of bone defects.

## 1. Introduction

Besides supporting the human body, bone tissue regulates blood pH, acts as a mineral source, and generates hematopoietic stem cells and Mesenchymal Stem Cells (MSCs) [1].

Today, tissue engineering represents a novel approach to repair bone-tissue defects in oral, orthopedic, and maxillofacial surgery and it has as principal actors: i) the cells and ii) the scaffold [2].

Scaffolds material are three-dimensional (3D) structures used in tissue engineering that mime the role of extracellular matrix and provide a mechanical support for cells. Moreover 3D biomaterials are also able to induce specific protein synthesis, stimulate some cellular activity, facilitate the cell adhesion and can stimulate the bone tissue formation in vivo. An appropriate scaffold should be osteogenic, i.e. able to induce the formation of bone tissue, osteoinductive, i.e. able to enroll MSCs derived from human tissues, and finally osteoconductive, i.e., able to support the growth of the bone tissue. The latter aspect of the scaffold properties is focused on the chemical composition and on the spatial geometric model qualified to provide to endothelial cells migration and sprouting of new vessels. 

To date, bone tissue regeneration strategies lack an approach that effectively provides an osteogenic and angiogenic environment conducive to bone growth. In fact in the bone formation, in skeletal development or in osseointegration process the interaction between osteogenesis and angiogenesis plays a fundamental role. Recent literature has investigated the effect of coculture in bone regeneration. For example in the coculture of mesenchymal cells and endothelial cells or in the coculture of human mesenchymal stem cells and human umbilical vein endothelial cells the bone regeneration process has been promoted [3].

MSCs derived from oral tissues are easy to isolate and collect. Human Periodontal Ligament Stem Cells (hPDLSCs) are mesenchymal stem cells that showed the classical fibroblast-like phenotype, the ability to differentiate into osteogenic, adipogenic and chondrogenic lineages, other than the positivity for stemness surface markers and the negativity for hematopoietic surface molecules [4]. The in vivo use of hPDLSCs avoid the immunological reaction, they also mediate their therapeutic effects through paracrine signalers by the release of extracellular vesicles [5,6]. Moreover, hPDLSCs combined with different biocompatible 3D scaffold demonstrated the ability to enhance the in vivo bone formation, in particular with collagen membranes and have shown to be induced toward an endothelial differentiation [2,7].

Native bone tissue is characterized by an abundant vascular network that is of the upmost importance for the diffusion of oxygen and nutrient transport—essential elements for cell tissue homeostasis. Recent literature indicates that in bone tissue engineering, human MSCs that are able to differentiate as osteoprogenitor cells and endothelial cells (ECs), playing a vital roles in bone regeneration [8,9]. Furthermore, the ERK/MAPK transduction signaling plays an important role in the bone tissue formation and some mutations in this mechanism are related to some human skeletal syndromes, including Noonan, Costello, and cardio-facio-cutaneous syndromes [10,11].

The aim of the present study is focused on the functionalization, in vitro, of a bovine pericardium collagen membrane with hPDLSCs and E-hPDLSCs coculture to obtain an improvement of healing process in terms of bone regeneration and neovascularization when the living construct is implanted in vivo.

## 2. Materials and Methods

### 2.1. Ethics Statement

The Medical Ethics Committee of G. d’Annunzio University, Chieti, Italy approved the present research (n° 266/17.04.14). Before enrollment, patients signed the informative consent form. The Department of Medical, Oral and Biotechnological Sciences and the Laboratory of Stem Cells and Regenerative Medicine are certified according to the quality standard International Organization for Standardization (ISO) 9001:2008 (certificate n° 32031/15/S).

### 2.2. hPDLSCs Isolation and Culture

Human PDLSCs were isolated from human periodontal ligament biopsies collected from healthy patients as previously reported [12]. During routinely clinical practice, tooth extraction for orthodontic purpose, the periodontal ligament tissue has been separated from the root. Biopsies were washed with Phosphate Buffered Saline (PBS; Lonza, Basel, Switzerland), were reduced in a small fragments and then were placed in a Petri dish with TheraPEAK™MSCGM-CD™ Bullet Kit serum free, chemically defined medium for the growth of human MSCs (Lonza) at 37 °C. Every three days the medium wash change with a fresh one. After 2 weeks of culture, cells spontaneously migrating from the cultured tissue. At 80% of confluence cells were trypsinized using Trypsin (LiStar Fish, Milan, Italy), and then subcultured until passage 2 (P2). For all performed experiments cells at P2 have been used.

### 2.3. hPDLSCs Characterization

Evaluation of hPDLSCs phenotype was conducted by flow cytometry, as previously described [6]. Briefly, 2.5 × 10^5^ cells were incubated for 30 min with following antibodies: anti-CD44-FITC, anti-CD105-FITC, anti-CD29-PE, and anti-CD45-FITC (Ancell Corporation, Bayport, MN, USA); anti-CD14-FITC (Miltenyi Biotec, Bergisch Gladbach, Germany); OCT3/4-PE, CD73-PE, SOX2-Alexa488, SSEA4-FITC, CD90-FITC (Becton Dickinson, San Jose, CA, USA), and CD34-PE (Beckman Coulter, Fullerton, CA, USA). After incubation with appropriate secondary antibodies, fixation in 1 mL PBS 0.5% paraformaldehyde and washing, cells were analyzed using a FACStar-plus flow cytometry system and the FlowJo™ software v10.0.7 (TreeStar, Ashland, OR, USA).

hPDLSCs at P2 were fixed with paraformaldehyde, stained with toluidine blue and observed with an inverted light microscopy Leica DMIL (Leica Microsystem, Milan, Italy). Images captured using a Nikon digital camera Digital Sight. For osteogenic and adipogenic differentiation, hPDLSCs were incubated in MSCGM-CD (Lonza) medium with osteogenic supplements and in adipogenesis induction/maintenance medium (Lonza), respectively. Both the mesengenic differentiation were confirmed by means of colorimetric assay as previously described. Osteogenic and adipogenic markers were evaluated by real-time polymerase chain reaction (PCR) according to Diomede et al., (2017) [6] expression of runt-related transcription factor 2 (RUNX2) and alkaline phosphatase (ALP) was evaluated after 7 days in osteogenic differentiated culture. The expression of fatty acid binding protein 4 (FABP4) and peroxisome proliferator-activated receptor γ (PPAR γ) were analyzed after 28 days of adipogenic differentiation culture. Commercially available TaqMan Gene Expression Assays (RUNX-2 Hs00231692_m1; ALP Hs01029144_m1; FABP4 Hs01086177_m1; PPAR2 Hs01115513_m1; ACAN Hs00153936_m1, Thermo Fisher, Life Tech, Monza, MB, Italy) and the TaqMan Universal PCR Master Mix (Thermo Fisher) were used according to standard protocols. Beta-2 microglobulin (B2M Hs99999907_m1) (Applied Biosystems, Foster City, CA, USA) was used for template normalization.

### 2.4. hPDLSCs Endothelial Differentiation

To induce the endothelial differentiation on cells were reached at 50–60% of confluency. Human PDLSCs were cultured with Endothelial Growth Medium (EGM-2; Lonza) composed by Endothelial Basal Medium-2, growth supplements (containing hydrocortisone, human Fibroblast Growth Factor (hFGF-b), R3-Insulin-like Growth Factor-1 (R3-IGF-1), ascorbic acid, human Epithelial Growth Factor (hEGF), GA-1000, heparin), 5% FBS and 50 ng/mL of Vascular Endothelial Growth Factor-165 (VEGF-165) (EGM-2 Bullet Kit; Lonza). Cells were maintained under differentiation conditions for 10 days. 

### 2.5. Analysis of CD31 and CD34 Expression

hPDLSCs and E-hPDLSCs were used for the phenotype analysis as previously reported by Pizzicannella et al. 2018 [13]. Human PDLSCs were stained using monoclonal antibody for CD31 and CD34 PE conjugated (Miltenyi Biotech, Bergisch Gladbach, Germany). Experiments were conducted in triplicate.

### 2.6. Tube Formation Test

Cultrex® Basement Membrane Extract (Trevigen Inc., Gaithersburg, MD, USA) at a concentration of 300 μL/well was used to cover the 12-well culture plates in order to perform the tube formation assay. Twelve-well culture plates were pretreated with Cultrex for 30 min, after matrix solidification, hPDLSCs and E-hPDLSCs were seeded at a density of 2 × 10^5^ cells. Tubular structures were evaluated after 4 h of culture at light microscopy (DMIL, Leica Biosystem, Milan, Italy). 

### 2.7. Scaffold Material

The BioRipar® (BioR; Assut Europe SpA, Magliano dei Marsi, AQ, Italy) is a medical device from bovine pericardium derived collagen membrane. The manufacturing process provided the inactivation of pathogens, the complete destruction and removal of cells without altering collagen 3D structure and biomechanical properties. Purified pericardium, composed by type I Collagen and Elastin, represented a new tool for fibroblasts growth and new blood vessel formation. Sterile scissors were used to cut the membrane in small piece size. PBS (Lonza) was used to rehydrate the BioR membrane before use.

### 2.8. Cells Cultured on Scaffold Material

10 × 10^5^ hPDLSCs, 10 × 10^5^ E-hPDLSCs and the coculture of hPDLSCs + E-hPDLSCs in the ratios of 1:1 were placed on BioR membrane and maintained in Dulbecco's Modified Eagle Medium (DMEM; Lonza) for two weeks before the following experiments.

### 2.9. Immunofluorescence Analysis

Human PDLSCs and E-hPDLSCs were processed for immunofluorescence detection as previously described by Pizzicannella et al. [13]. Primary monoclonal antibodies anti-human CD31 (1:300, mouse) (Dako, Agilent Technologies, Santa Clara, CA, USA), CD34 (1:200, mouse) (Dako) and Vascular Endothelial Growth Factor-A (VEGF-A) (1:200, mouse) (Santa Cruz Biotechnology, Santa Cruz, CA, USA) was used, followed by Alexa Fluor 568 conjugated goat anti-mouse as secondary antibodies (1:200) (Thermo Fisher) for 1 h at 37 °C. Alexa Fluor 488 phalloidin green fluorescence conjugate (1:200) (Thermo Fisher) was used to evaluate the cytoskeleton actin and TOPRO (1:200) (Thermo Fisher) was used to mark cell nuclei. Glass coverslips were placed face down on glass slides and mounted with Prolong antifade (Thermo Fisher) [14].

To evaluate the morphological aspects of hPDLSCs, E-hPDLSCs and the coculture of hPDLSCs + E-hPDLSCs were labeled with different cell tracker: Human PDLSCs were incubated for 30 min with the green fluorescent cell linker, PKH67 (Sigma-Aldrich, Milan, Italy), and E-hPDLSCs were incubated for the same time with PKH26, red fluorescent lipophilic dye (Sigma-Aldrich). The PKH67-labeled hPDLSCs and the PKH26-labeled E-hPDLSCs were seeded on BioR membrane and then incubated for 2 weeks at 37 °C in 5% CO_2_ and air [15].

Zeiss LSM800 META confocal microscopy (CLSM, Zeiss, Jena, Germany) has been used to evaluate stained samples through a Plan-NEFLUAR oil-immersion objective.

### 2.10. Alizarin Red S Assay

hPDLSCs, E-hPDLSCs and hPDLSCs + E-hPDLSCs cultured with and without BioR at 8 × 10^3^ cells/cm^2^ were used to evaluate the mineralization process. Alizarin Red S (ARS) staining has been performed after 2 weeks of culture to evaluate the cell ability to form calcium deposition. All samples before the fixation process were washed with PBS (Lonza) and then were fixed in 10% (v/v) of formaldehyde (Sigma-Aldrich) solution for 30 min. Samples were washed with dH_2_O and the were stained with Alizarin Red S solution as previously reported. To evaluate the colorimetric assay at spectrophotometer (Synergy HT, BioTek, Bad Friedrichshall, Germany) samples were processed as previously described [6]. 

### 2.11. RNA Isolation and Quantitative Real-Time PCR

RNA derived from hPDLSCs, E-hPDLSCs and hPDLSCs + E-hPDLSCs cultured with and without BioR was isolated with the RNeasy Plus Universal Mini Kit (Qiagen, Valencia, CA, USA, https://www.qiagen.com). Quantitative PCR was performed as previously described by Diomede et al. [16]. VEGFA (Hs00900055_m1, Thermo Fisher), RUNX2 (Hs01047973_m1, Thermo Fisher), MAPK3/ERK1/2 (Hs00385075_m1, Thermo Fisher) and COL1A1 (Hs00164004_m1, Thermo Fisher) were used in the experiment. For internal control Beta-2 microglobulin (B2M, Hs99999907_m1; Applied Biosystems) has been used. Duplicates were set up for each sample and the mRNA expression was analyzed by the comparative 2-ΔΔCt relative quantification method.

### 2.12. Western Blot Analysis

Proteins derived from all samples hPDLSCs, E-hPDLSCs and hPDLSCs + E-hPDLSCs cultured with and without BioR were separated on Sodium Dodecyl Sulphate-Polyacrylamide Gel Electrophoresis (SDS-PAGE) and transferred to nitrocellulose sheets using a semidry blotting apparatus as previously described [17]. The saturation process was obtain after placed sheets in blocking buffer (1x TBS, 5% milk, 0.1% Tween-20) for 2 h, then were incubated overnight at 4 °C in blocking buffer containing different primary antibodies. In this experiment COL1A1 (1:2000, rabbit) (OriGene), VEGF-A (1:2000, rabbit) (Santa Cruz Biotechnology), ERK1/2 (1:1000, rabbit) (OriGene), RUNX-2 (1:1000, mouse) (Santa Cruz Biotechnology) and β-Actin (1:750, mouse) (Santa Cruz Biotechnology) were used as primary antibodies. Then samples were incubated with peroxidase-conjugated secondary antibody diluted 1:1000 in 1x Tris-Buffered Saline (TBS), 2.5% milk, 0.1% Tween-20 [18]. Electrochemiluminescence (ECL) method has been used to evaluate the specific protein bands by means Alliance 2.7 (UVItec Limited, Cambridge, UK) [19].

### 2.13. Osteocalcin Immunoassay

To quantify the osteocalcin levels in the supernatant derived from hPDLSCs, E-hPDLSCs and hPDLSCs + E-hPDLSCs cultured with and without BioR, the Metra TM osteocalcin immunoassay (Quidel Corporation, San Diego, CA, USA) was used. Normalization was related to the cell number.

### 2.14. Statistical Analysis

Prism 4 GraphPad package (Prism 4 GraphPad software, 4.0, San Diego, CA, USA) has been use to evaluate the statistical significance. ANOVA and Tukey’s post hoc analysis were used as statistical tests (p < 0.05).

## 3. Results

### 3.1. hPDLSCs Characterization

**Figure 1:**
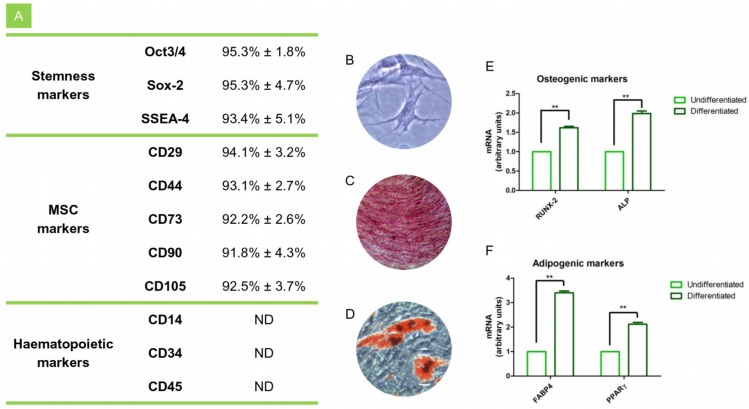
hPDLSCs characterization. (**A**) Cytofluorimetric analysis of hPDLSCs. (**B**) Plastic adherent hPDLSCs stained with toluidine blue solution. (**C**) Osteogenic differentiated hPDLSCs stained with Alizarin Red solution. (**D**) Adipogenic differentiated hPDLSCs stained with oil red solution. (**E**) RT-PCR of osteogenic related markers in undifferentiated and differentiated cells. (**F**) RT-PCR of adipogenic related markers in undifferentiated and differentiated cells. **p < 0.01. Scale bar: 10 µm.

In order to confirm hPDLSCs phenotype, flow cytometry analysis was conducted on stem cells at the second passage. Ex vivo expanded hPDLSCs showed positivity for Oct3/4, Sox-2, SSEA-4, CD29, CD44, CD73, CD90, and CD105. On the contrary, the hematopoietic stem cell markers as CD14, CD34, and CD45 were negative (Figure 1A). Cells morphological features were observed under light microscopy, hPDLSCs adhered to the plastic substrate and showed a fibroblast like morphology (Figure 1B). After induction period of osteogenic and adipogenic commitment cells were stained with Alizarin Red and adipo oil red solution, respectively. In osteogenic differentiated cells calcium deposits were evident (Figure 1C), as well as the lipid droplets were stained in red at cytoplasmic level in cells committed to adipogenic lineage (Figure 1D). RT-PCR confirmed the qualitative data in differentiated cells. In fact the gene expression of ALP, RUNX2, FABP4 and PPARγ showed high level in differentiated cells when compared to the undifferentiated (Figure 1E,F).

### 3.2. Isolation of hPDLSCs and Characterization of E-hPDLSCs

**Figure 2:**
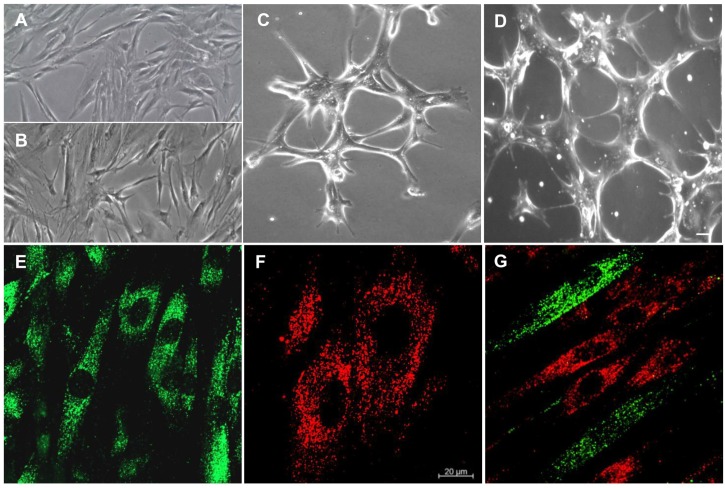
Morphological analyses. (**A**) Undifferentiated hPDLSCs observed at light microscopy. (**B**) E-hPDLSCs observed at light microscopy. (**C**,**D**) E-hPDLSCs cultured on Cultrex® observed at light microscopy. (**E**) Undifferentiated hPDLSCs evaluated under CLSM (PKH67, green fluorescence). (**F**) E-hPDLSCs evaluated under at CLSM (PKH26, red fluorescence). (**G**) Coculture of hPDLSCs and E-hPDLSCs. Scale bar: 20 µm. Magnification: 10× (**A**–**D**). Magnification: 20× (**E**–**G**).

Plastic-adherent hPDLSCs showed a long cytoplasmic processes shape with numerous contact with neighbouring cells (Figure 2A). After a differentiation period, the cells changed their appearance. E-hPDLSCs showed more contacts among other cells (Figure 2B). In fact, E-hPDLSCs placed onto Cultrex® substrate showed a change in their common morphological aspect and they also started to take many contacts forming tubular structures (Figure 2C,D). PKH67 (green fluorescence) stained-hPDLSCs observed under CLSM showed the typical fibroblast-like morphology (Figure 1E), while E-hPDLSCs, stained with PKH26 (red fluorescence) showed a different morphological aspect with short structure (Figure 2F). Immunofluorescence evaluation of the coculture hPDLSCs + E-hPDLSCs showed the presence of both cellular type, is clearly visible the different cell shape, hPDLSCs has been marked in green, while E-hPDLSCs has been tracked in red (Figure 1G).

### 3.3. CD31, CD34 and VEGFA Expression

**Figure 3:**
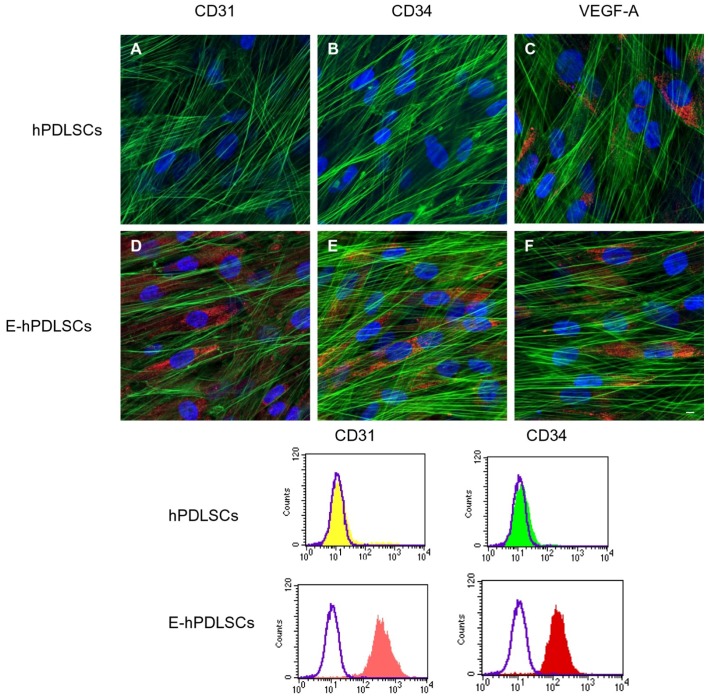
Immunofluorescence analyses. (**A**) Undifferentiated hPDLSCs showed a negative expression for CD31. (**B**) Undifferentiated hPDLSCs showed a negative expression for CD34. (**C**) Undifferentiated hPDLSCs showed a slightly positive expression for Vascular Endothelial Growth Factor-A (VEGF-A). (**D**) E-hPDLSCs showed a strong positive expression for CD31. (**E**) E-hPDLSCs showed a strong positive expression for CD34. (**F**) E-hPDLSCs showed a strong positive expression for VEGF-A. Scale bar: 10 µm. Magnification: 20×. Green fluorescent: cytoskeleton actin; Blue fluorescent: nuclei; Red fluorescent: specific markers. Cytofluorimetric analysis of the expression of CD31 and CD34 in hPDLSCs and in E-hPDLSCs confirm the immunofluorescence qualitative results.

Human PDLSCs at basal condition were negative for CD31 and CD34, while showed a slightly positivity for VEGF-A (Figure 3A–C). While, E-hPDLSCs showed a strong positivity for the above mentioned markers (Figure 3D–F). The immunofluorescence data were confirmed by cytofluorimetric results that show the positivity for CD31 and CD34 in E-hPDLSCs, while the hPDLSCs were negative for the same markers (Figure 3).

### 3.4. Cell Culture on Scaffold Material

**Figure 4:**
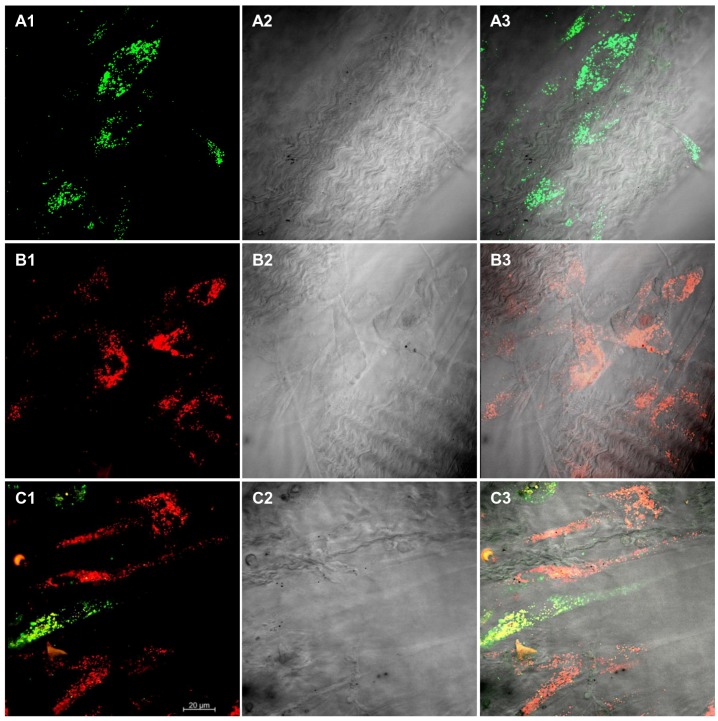
Cell and 3D scaffold interaction. (**A1**) hPDLSCs, stained with PKH67 (green fluorescence), cultured on BioR showed a fibroblast like morphology. (**A2**) Light transmission image of BioR membrane (grey scale). (**A3**) Merged image of above mentioned channels. (**B1**) E-hPDLSCs, stained with PKH26 (red fluorescence), cultured on BioR. **(B2**) Light transmission image of BioR membrane (grey scale). (**B3**) Merged image of above mentioned channels. (**C1**) Coculture of hPDLSCs and E-hPDLSCs cultured on BioR showed the co-presence of two different cellular types with green and red fluorescence. (**C2**) Light transmission image of BioR membrane (grey scale). (**C3**) Merged image of above mentioned channels. Scale bar: 20 µm; Magnification: 20×.

CLSM observations showed hPDLSCs, E-hPDLSCs and hPDLSCs + E-hPDLSCs cultured on BioR membrane (Figure 4). PKH67 stained hPDLSCs demonstrated an elliptical shape similar to fibroblasts stained in green (Figure 4A1–A3), while E-hPDLSCs cultured on BioR showed a thin and short shape morphology (Figure 4B1–B3). Figure 3 section C3 showed the coculture of hPDLSCs + E-hPDLSCs on the BioR membrane. Figure 3 sections A4, B4 and C4 showed the measurements of long and short diameters, corresponding to the major and minor axes of an ellipse evaluated at CLSM pictures. In particular hPDLSCs showed an aspect ratio near 0.20, while E-hPDLSCs showed a ratio near 0.60, indicating that exist a difference among long and short diameters.

### 3.5. Osteogenic Differentiation

**Figure 5:**
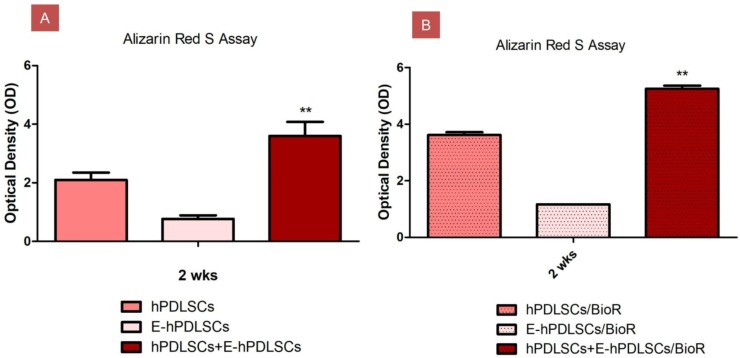
Osteogenic differentiation detection. Alizarin Red S assay. Bar chart of the quantification of Alizarin Red S staining read at spectrophotometer. (**A**) Colorimetric assay to detect the osteogenic differentiation of cells; (**B**) Colorimetric assay to detect the osteogenic differentiation of cells cultured with BioR; ** *p* < 0.01.

Obtained results of the Alizarin Red S staining were evaluated using spectrometric analysis after 2 weeks of culture. hPDLSCs, E-hPDLSCs and hPDLSCs + E-hPDLSCs cultured without collagen membrane showed a different intensity staining when compared to the same groups cultured with BioR. hPDLSCs + E-hPDLSCs cultured with BioR showed the best results when compared to the single culture maintained with biomaterial and with hPDLSCs + E-hPDLSCs cultured on a plastic substrate (Figure 5).

### 3.6. Gene Expression

**Figure 6:**
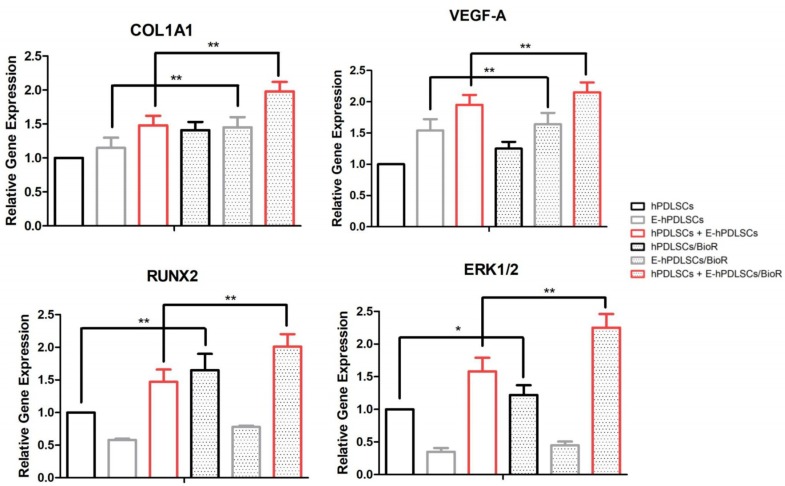
RT-PCR. Graph bars showed the gene expression of COL1A1, VEGF-A, RUNX2 and ERK1/2 in hPDLSCs, E-hPDLSCs and hPDLSCs + E-hPDLSCs cultured with and without BioR. ** *p* < 0.01 and * *p* < 0.05.

RT-PCR performed on hPDLSCs, E-hPDLSCs and hPDLSCs + E-hPDLSCs cultured with and without BioR showed an up regulation of COL1A1, VEGF-A, RUNX2 and ERK1/2. hPDLSCs + E-hPDLSCs samples showed an high expression of all considered markers when compared to hPDLSCs or E-hPDLSCs. The best results have been obtained in hPDLSCs + E-hPDLSCs cultured on BioR when compared to hPDLSCs/BioR and with the coculture sample maintained without the collagen membrane. The level of mRNA of the same studied gens in E-hPDLSCs/BioR showed a slow increase when compared to the control sample (Figure 6).

### 3.7. Western Blot Analysis

**Figure 7:**
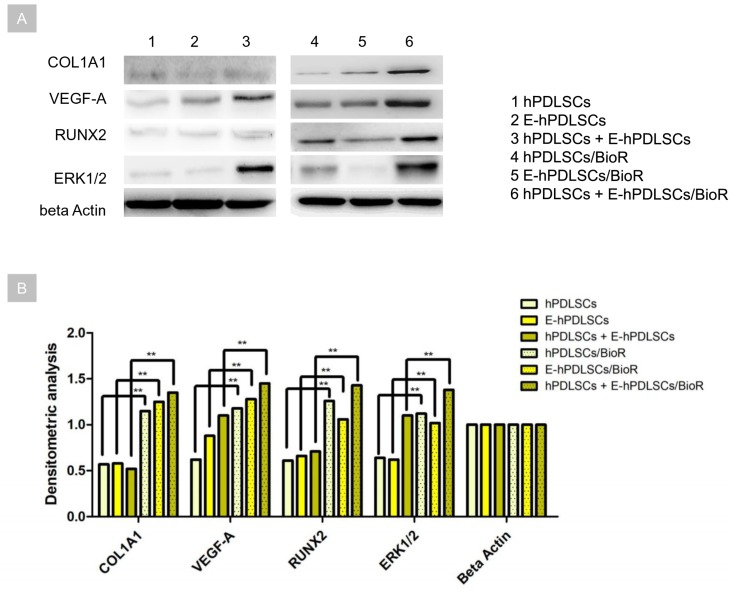
Western blot. (**A**) Protein levels specific band. Beta actin has been used as housekeeping protein. (**B**) Graph showed the specific band densitometric levels. ** *p* < 0.01.

The proteins level was evaluated in hPDLSCs, E-hPDLSCs and hPDLSCs + E-hPDLSCs cultured with and without BioR. The specific bands were analysed for their densitometry analysis, the related histogram showed an upregulation of COL1A1, VEGF-A, RUNX2 and ERK1/2 in hPDLSCs + E-hPDLSCs cultured on BioR when compared with hPDLSCs and E-hPDLSCs, respectively. Specific bands of RUNX2 and ERK1/2 have been down-expressed in E-hPDLSCs when compared with hPDLSCs maintained with BioR. At the same time hPDLSCs, E-hPDLSCs and hPDLSCs + E-hPDLSCs cultured without BioR showed a low expression of COL1A1, VEGF-A, RUNX2 and ERK1/2 when compared with cells cultured with collagen membrane (Figure 7).

### 3.8. Osteocalcin Modulation

**Figure 8:**
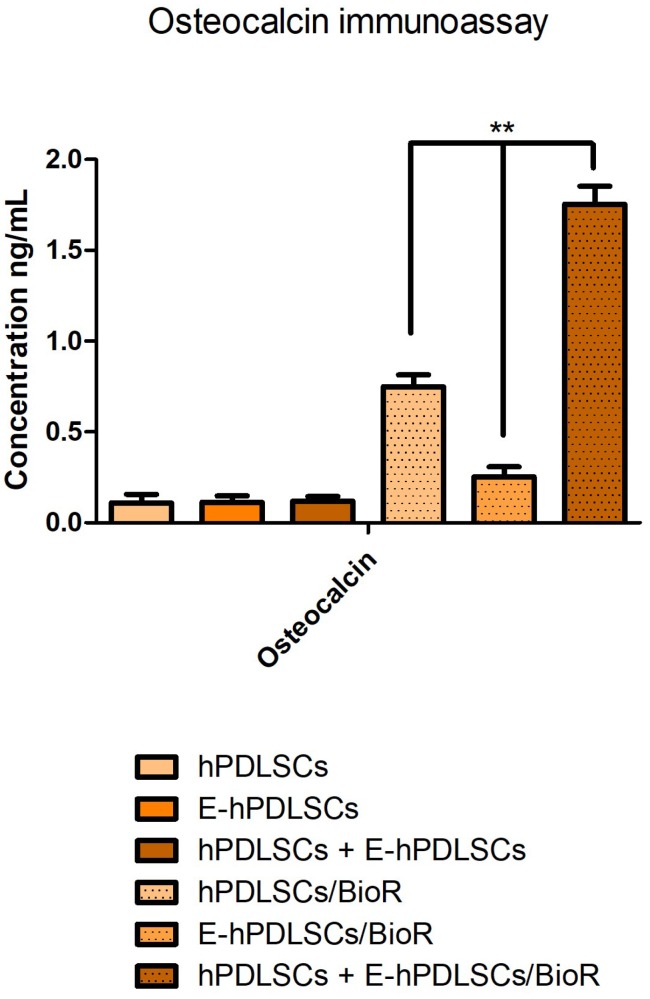
Osteocalcin immunoassay. Graph showed the osteocalcin level measured in supernatant after two weeks of culture in hPDLSCs, E-hPDLSCs and hPDLSCs + E-hPDLSCs cultured with and without BioR. Experiments were performed in triplicate. ** *p* < 0.01.

The histogram of the immunoassay test showed an increase level of osteocalcin in the supernatant of differentiated hPDLSCs + E-hPDLSCs cultured on BioR in comparison with hPDLSCs and E-hPDLSCs incubated with the collagen membrane. Cells cultured without BioR membrane showed low level of osteocalcin measured in the supernatants when compared to the same group cultured with membrane (Figure 8).

## 4. Discussion

During embryonic development, two significant models of bone formation are present: intramembranous ossification and endochondral ossification, in both cases benefiting from a vascular network. In fact, bone tissue presents an extensive network of blood vessels consuming almost 10–15% of resting cardiac output [20]. Blood vessels with their particular spatial structure provide a good exchange of gas and nutritive macromolecules for bone tissue and for the bone marrow compartments. Bone tissue vascularization is furnished by cortical region arteries that linking with the medullary sinusoids. Scaffold materials play a crucial role in the bone tissue engineering field. Scaffolds should be made by a biocompatible materials, with a three dimensional (3D) structures that permit the cell adhesion in order to favour in vivo the bone regenerative process that is a balanced mechanism shared among the cell interactions and the biomechanical properties. [21,22,23,24]. Regenerative medicine is a field of tissue engineering that link different fundamental components: cell biology and biomaterials. Biomaterials are key factors to obtain a good results in term of bone regeneration, in fact the 3D scaffold provide structure that permit cell adhesion and their support [25]. A bone analogous scaffold should contain components able not only to promote osteogenesis but also to support angiogenesis in order to prevent hypoxia-induced cell death [26]. Today, oral tissues are considered a wide studied source of mesenchymal stem cells (MSCs) that can be used in regenerative medicine [27,28], in particular oral-derived MSCs. They can be loaded on several different scaffold materials in order to enhance the bone regenerative process [15,29]. Human PDLSCs release stromal cell-derived factor-1 (SDF1) [13] that can promote the cell migration and improve angiogenesis and healing process [30]. One more main advantage of using hPDLSCs is the production of angiogenic cytokines that can contribute to angiogenesis stimulation via miR-210 [13]. Human PDLSCs are easy to collect, in fact they are located in the periodontal tissue, a soft tissue that provide a mechanical stability to the tooth [31]. Moreover, hPDLSCs showed a positivity for the expression of perivascular cell markers, as neural/glial antigen 2, alpha-smooth muscle actin, platelet-derived growth factor receptor ß and CD146 [13].

Stem cells with neural crest origin are able to generate the endothelial tissues, in particular the stem cells derived from the dental pulp, the periodontal ligament and the gingival tissue showed a strong angiogenic potential [2,32,33,34]. In the present study, a 3D living construct, constituted by collagen membranes as substrate, in association with hPDLSCs and E-hPDLSCs, was evaluated. Human PDLSCs, induced to endothelial differentiation, were positive for CD31, CD34 and VEGF-A, other than to show the ability to form capillary-like tubes network after seeding on Cultrex®. Endothelial stem cells differentiated from periodontal ligament tissues can be considered a good alternative source to use for the endothelial regeneration [13]. 

Previous studies have been demonstrated that cell proliferation can be increased in the coculture system, for example in the coculture of endothelial cells (ECs) and precursor bone cells [35]. Moreover, Gershovich et al. reported that the osteogenesis can be enhanced in the coculture of MSCs and Human Umbilical Vein Endothelial Cells (HUVEC) seeded onto 3D scaffold [36].

The aim of the present study is focused on the regenerative potential of hPDLSCs and E-hPDLSCs co-culture loaded on pericardium collagen membrane in order to enhance in vitro the bone regeneration process.

Bone-regeneration process was stimulated from hPDLSCs seeded onto BioR membrane, suggesting that endothelial cells could be responsible for a more rapid evolution in the newly formed bone. Obtained results were confirmed by the demonstration of higher level of RUNX2 and COL1A1 expression other than the mineralization process visualized through Alizarin Red S staining. RUNX2 is a transcription factor involved in osteoblast differentiation and skeletal morphogenesis, in particular its role is related to the BMPs differentiation induction [37]. In the mineralized bone matrix osteocalcin, non-collagenous protein, plays a key role as an inductor of osteogenesis in vitro. In our experimental model osteocalcin was higher upregulated in coculture seeded on BioR.

The ERK/MAPK transduction signal mediates the intracellular signaling activity regulated by several different factors as fibroblast growth factors (FGFs), bone morphogenetic proteins (BMPs) and tumor growth factors (TGFs) [38] and it is critical for VEGF-A/VEGFR2-induced differentiation of adipose mesenchymal stem cells into ECs [39]. In our experimental model the upregulation of VEGF-A in the living construct, composed by hPDLSCs + E-hPDLSCs/BioR, could contribute to enhance an osteogenic microenvironment for the periodontal ligament stem cells through the ERK1/2 activation.

## 5. Conclusions

Considered the limitation of this study, we retain that hPDLSCs + E-hPDLSCs/BioR seemed then to be able to influence skeletal segment regeneration representing a simple and effective strategy for improving stem-cell therapy.

## Abbreviations

MSCs	Mesenchymal Stem Cells
hPDLSCs	human Periodontal Ligament Stem Cells
E-hPDLSCs	Endothelial differentiated human Periodontal Ligament Stem Cells
BioR	Collagen membrane
VEGF-A	Vascular Endothelial Growth Factor-A
3D	Three-Dimensional
ERK1/2	Extracellular signal-Related Kinase-1/2
MAPK	Mitogen-Activated Protein Kinase
RUNX2	Runt-related transcription factor-2
COL1A1	Collagen 1A1
EDTA	EethyleneDiamineTetraacetic Acid
hEGF	human Epithelial Growth Factor
hFGF	human Fibroblast Growth Factor
HUVEC	Human Umbilical Vein Endothelial Cells
SDF1	Stromal cell-Derived Factor-1
ECs	Endothelial Cells
